# The ALMT Family of Organic Acid Transporters in Plants and Their Involvement in Detoxification and Nutrient Security

**DOI:** 10.3389/fpls.2016.01488

**Published:** 2016-10-04

**Authors:** Tripti Sharma, Ingo Dreyer, Leon Kochian, Miguel A. Piñeros

**Affiliations:** ^1^Centro de Bioinformática y Simulación Molecular, Facultad de Ingeniería, Universidad de Talca, TalcaChile; ^2^Robert W. Holley Center for Agriculture and Health, United States Department of Agriculture–Agricultural Research Service, Cornell University, Ithaca, NYUSA

**Keywords:** anion channel, ALMT, aluminum tolerance, nutrient transport, malate transport, citrate transport, review

## Abstract

About a decade ago, members of a new protein family of anion channels were discovered on the basis of their ability to confer on plants the tolerance toward toxic aluminum ions in the soil. The efflux of Al^3+^-chelating malate anions through these channels is stimulated by external Al^3+^ ions. This feature of a few proteins determined the name of the entire protein family as Aluminum-activated Malate Transporters (ALMT). Meanwhile, after several years of research, it is known that the physiological roles of ALMTs go far beyond Al-detoxification. In this review article we summarize the current knowledge on this transporter family and assess their involvement in diverse physiological processes.

## Introduction

### Organic Acids in Plants – Production, Importance, and Function

Organic acids play pivotal roles in plant primary metabolism. These acids are mainly produced and involved in central metabolic pathways, such as the tricarboxylic acid cycle, C3-, C4-, and CAM-photosynthesis and, to lesser extent, in the glyoxylate cycle in plants. Organic acids, such as malate, fumarate, lactate, and citrate, are of fundamental importance at the cellular level for several biochemical pathways, including energy production, formation of precursors for amino-acid biosynthesis, and at the whole plant level in modulating adaptation to the environment (Igamberdiev and Eprintsev, 2016). They are involved in stomatal function, phosphorous acquisition, aluminum tolerance, and temporary carbon storage, interchange of reductive power among subcellular compartments, pH regulation, and the response to biotic and abiotic stresses (Meyer et al., 2010a). Some organic molecules also function as signaling molecules, not only as allosteric regulators of many key enzymes, but also as modulators of gene expression (López-Bucio et al., 2000).

Research carried out in the past two decades revealed the importance of organic acid exudation for plants encountering/tolerating metal and nutritional stress at the root-soil interface. The roots of rape (*Brassica napus*), for instance, excrete citric and malic acids into the rhizosphere and solubilize P from rock phosphate (Hoffland et al., 2006). Root exudation of citrate may play an important role in supplying Fe to dicotyledonous plants (Brown, 1978). Organic acids have also been found to modulate nitrate uptake. Malate moves down the phloem, accumulates in the root, and stimulates the uptake of nitrate by the roots of intact soybean plants (Touraine et al., 1992). Plants growing in alkaline soils, where calcium is abundant, often reduce the excess of cellular calcium by precipitating it in the form of calcium oxalate in vacuoles of roots, stems, leaves, flowers, fruits, and seeds (Webb, 1999). Likewise, the most ubiquitous tolerant mechanism in the plant kingdom is the exclusion of Al from the root apex via root exudation of organic acids.

### Historical Perspective – Al Tolerance as a Gateway for the Discovery of a New Family of Anion Transporters

**FIGURE 1:**
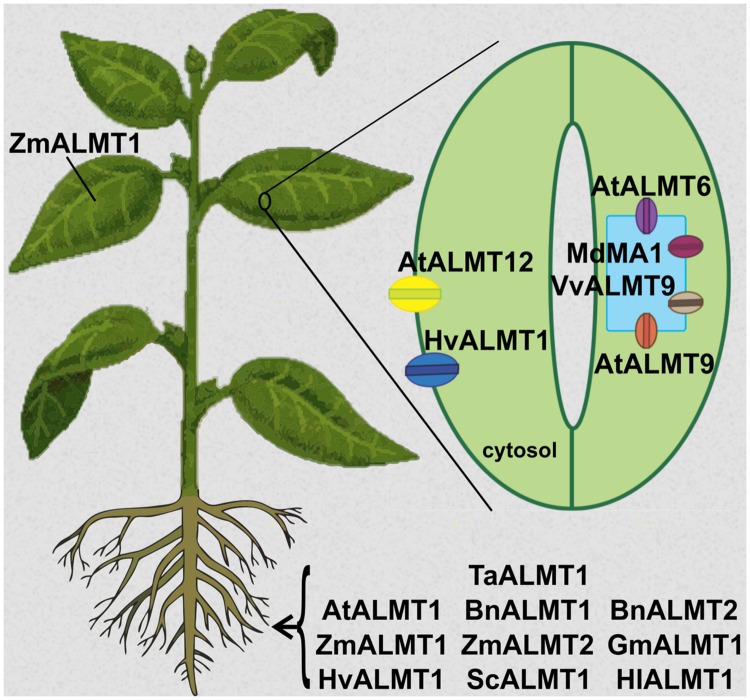
**Tissue localization of Aluminum-activated Malate Transporters (ALMTs) from different plant species.** AtALMT1, BnALMT1, BnALMT2, GmALMT1, HlALMT1, ScALMT1, TaALMT1 are plasma membrane channels expressed in roots and are functionally involved in providing Al^3+^ tolerance to the plant. ZmALMT1 is found at the plasma membrane of cells throughout the plant, while ZmALMT2 is localized at the plasma membrane in root cells; both are also involved in anion homeostasis. In guard cells, AtALMT6 and AtALMT9 are targeted to the tonoplast, while AtALMT12 and HvALMT1 are localized at the plasma membrane; all play crucial roles in guard cell movement and thus stomatal opening and closure. HvALMT1 is expressed in guard cells and in root cells, where it is involved in turgor regulation in expanding cells. MdMA1 and VvALMT9 are tonoplast channels with expression in guard cells; they are involved in fruit flavor development in apple and grapes, respectively.

Electrophysiological approaches in the 1990’s and early 2000 established the existence and early biophysical characterization of a variety of predominantly plasma membrane- and tonoplast-localized anion channels in plant cells (Tyerman, 1992; Roberts, 2006). Contrary to expectations, the molecular identity of most of these anion channels remained elusive, when the genome of *Arabidopsis thaliana* became accessible. This was predominantly due to the fact that the identified homologs of animal counterparts were mainly constituted by members of the CLC channel family (Hechenberger et al., 1996; Jentsch et al., 1999; Chen, 2005; Jentsch, 2008). Consequently, seven CLC homologs were identified in the *Arabidopsis* genome, and have been shown to localize to cellular endomembranes (tonoplast, golgi, and chloroplast). As some of their animal counterparts, they functionally encode H^+^/Cl^-^ exchangers [reviewed by (Barbier-Brygoo et al., 2011)], but they are also involved as nitrate/proton antiporters in the nitrate accumulation in plant vacuoles (De Angeli et al., 2006). Paradoxically, the molecular identity of a new family of anion channels involved in a large variety of *in planta* roles, was revealed by studies in the field of Al-tolerance. Classical plant physiology studies provided evidence that several monocot and dicot plant species exclude phytotoxic Al^3+^ from entering the cells of the root tip by exuding di- and tri-carboxylic acids (e.g., citrate, malate, and/or oxalate), thereby chelating and immobilizing the soluble Al^3+^ at the root surface by forming stable, non-toxic complexes (Delhaize et al., 1993a,b; Ma et al., 1997). The thermodynamic nature of the transport, i.e., a large electrochemical gradient of organic acids from the cytosol to the apoplasm, suggested anion channels mediated the organic acid efflux. This prompted the implementation of electrophysiological techniques, the patch clamp technique for instance, to further characterize the transport process. With such an approach Ryan and coworkers (Ryan et al., 1997; Zhang et al., 2001) provided the first proof of an Al^3+^-dependent plasma membrane anion (malate selective) conductance in wheat root protoplasts from an Al-tolerant line. Evidence for the existence of similar types of channels in protoplasts from Al-resistant maize roots was reported soon after (Kollmeier et al., 2001; Piñeros and Kochian, 2001; Piñeros et al., 2002), further indicating that these single anion channels could be activated by Al^3+^ in outside-out excised membrane patches in the absence of additional cytosolic factors. The similarities between the transport and activation properties reported in these electrophysiological studies and the Aluminum-activated organic acid exudation observed in intact wheat and maize roots provided the first indication that this novel type of anion channel was likely underlying the Al-activated organic acid release observed at the whole root level. Further elucidation of the molecular nature of these transporters came from a subtractive hybridization approach, using a pair of near-isogenic wheat lines differing at a single Al tolerance locus. This approach led to the identification of the *TaALMT1* gene (formerly named *ALMT1*), the founder of the so called ALMT (Aluminum-activated Malate Transporters) family (Sasaki et al., 2004). Heterologous expression of this gene in roots of transgenic rice seedlings and tobacco suspension cells conferred an Al-dependent exudation of malate. Consistently, functional analysis in *Xenopus* oocytes indicated that this transporter mediated an inward current caused by a malate efflux, which strongly depended on the presence of extracellular Al^3+^. Transgenic barley (*Hordeum vulgare*) expressing TaALMT1 also showed an increase in Al-resistance in roots (Delhaize et al., 2004). Although TaALMT1 is functionally active in the absence of extracellular Al^3+^, this unique functional property of Al-mediated enhancement of transport activity (awkwardly referred to as Al-activation) is responsible for the sometimes misleading historical name comprising all members of this transporter family as ALMT. To date ‘Al activation’ has only been reported in a small subset of ALMTs members, while *in planta* the functions of a large number of members have been shown to be highly diverse and transcend beyond root Al-resistance responses. Soon after its discovery in wheat, Al tolerance-related studies led to the identification of *AtALMT1*, the first out of 14 *Arabidopsis* ALMT members to be functionally characterized, as well as the homologs *BnALMT1* and *BnALMT2* from rape, *GmALMT1* in soybean, and *ScALMT1* in rye. They all shared similar functional characteristics consistent with their involvement in mediating the organic acid exudation in Al-tolerance response in these plant species (Hoekenga et al., 2006; Ligaba et al., 2006, 2007). Likewise, *MsALMT1* from *Medicago sativa* and *HlALMT1* from the grass *Holcus lanatus* have been described as crucial genes involved in Al resistance (**Figure 1**) (Chen Q. et al., 2013; Chen Z.C. et al., 2013). Subsequent studies in relation to the role of the novel and emerging ALMT family in mediating Al-resistance responses led to the identification of the maize homolog ZmALMT1. However, although ZmALMT1 was shown to function as a plasma membrane transporter capable of mediating a selective anion efflux and influx, the gene expression data as well as biophysical transport characteristics provided the first indication in the literature that the *in planta* function of some members of this ALMT family might extend beyond Al tolerance to a variety of physiological processes (Piñeros et al., 2008b). Since then an increasing number of studies have clearly indicated that ALMTs underlie a variety of processes, including metal toxicity avoidance, mineral nutrition, ion homeostasis, turgor regulation, fruit quality, and guard cell function.

## Physiological Roles

### Root Abiotic Stress Responses – Adaptation to Acid Soils

Aluminum-activated Malate Transporters proteins play pivotal roles in the adaptation to acid soils. The main challenge of acid soils is the increased mobility of aluminum ions and their tendency to form highly stable complexes with phosphorus. Thus, a plant faces not only the toxicity of Al^3+^ ions but also a poor bioavailability of phosphate. By releasing organic acids both problems are tackled: The carboxylates chelate Al^3+^ ions and set phosphates free. However, the plant has to find a balance between the positive effects of organic acid release and the disadvantages of losing valuable carbon sources. Consequently, ALMT proteins are regulated at the transcriptional and functional level. The expression of *AtALMT1*, for instance, is under control of assorted signal inducers like abscisic acid (ABA) and indol-3-acetic acid (IAA) along with low pH and hydrogen peroxide (Kobayashi et al., 2013; Sukweenadhi et al., 2015). Elevated expression of AtALMT1 at low pH and in the presence of H_2_O_2_ coincides with the observation that Al treatment results in H_2_O_2_ accumulation in the root tips and in a reduction of the pH (Kobayashi et al., 2013). In soybean also phosphorus has been identified as signaling molecule (Liang et al., 2013). Root malate exudation appears to be critical for soybean adaptation to both Al toxicity and P deficiency on acid soils and the underlying channel GmALMT1 is coordinately regulated by pH, aluminum, and phosphorus.

### Guard Cell Regulation

Regulation of CO_2_ uptake and water loss in plants is achieved through the regulation of the stomatal aperture via osmotically driven guard cell movements (Kollist et al., 2014). Plasma membrane anion channels play an important role in stomatal movements by releasing anions and contributing to the depolarization of the guard cell plasma membrane (Barbier-Brygoo et al., 2011; Kollist et al., 2011; Roelfsema et al., 2012). Early electrophysiological studies in guard cells established the existence of two types of plasma membrane anion channels with distinct activation and deactivation kinetics, thereby named rapid (R)-type (QUAC) and slow (S)-type channels (SLAC; Hedrich et al., 1990; Linder and Raschke, 1992; Schroeder and Keller, 1992). Although less studied, malate permeable tonoplast channels were also identified in early studies (Pantoja et al., 1989).

**FIGURE 2:**
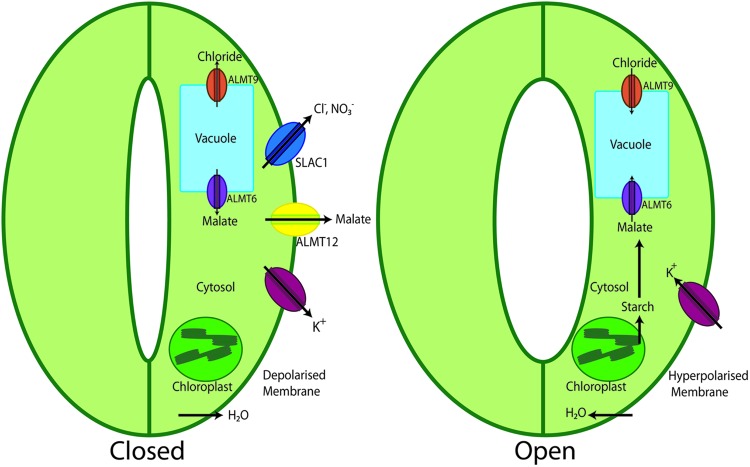
**Schematic representation of the role of ALMTs in stomatal opening and closure.** During stomatal closure K^+^ is released from the guard cell along with the release of malate via ALMT12 and Cl^-^ and NO_3_^-^ ions via SLAC1 channels. This loss of osmolytes culminates in loss of water from the cell resulting in guard cell shrinking and stomatal closure. On the contrary, during stomatal opening, K^+^ enters the cells, starch is degraded to malate balancing the charges. Upon increased malate concentrations it is transported to the vacuole by ALMT6. Besides, also ALMT9 is activated to facilitate the transport of Cl^-^ into the vacuole. This accumulation of solutes in the vacuole of guard cells decreases the water potential; water enters the cell causing the swelling of the guard cell, which finally results in stomatal opening.

Following the identification and characterization of the *Arabidopsis* root plasma membrane-targeted AtALMT1 channel (Hoekenga et al., 2006), studies evaluating the cellular localization of GFP-tagged AtALMT proteins, as well as promoter–GUS fusion experiments, indicated that two other channels of this protein family, AtALMT9 and AtALMT6, were localized to the tonoplast of guard cells, albeit not exclusively, proposing a role of these channels in vacuolar malate transport (**Figure 2**) (Kovermann et al., 2007; Meyer et al., 2011). Although *Atalmt9* KO plants were reported to show no visible phenotype, the malate flux across the membrane of vacuoles isolated from the *Atalmt9* KO appeared to be reduced compared to that recorded on wild type vacuoles (Kovermann et al., 2007). Consistently, heterologous expression of AtALMT9 in *Nicotiana benthamiana* leaves and *Xenopus* oocytes resulted in an enhancement of the malate current density across the mesophyll tonoplasts and oocyte plasma membrane, respectively. A more detailed analysis of AtALMT9 in its native environment, as well as after heterologous expression, however, has indicated that AtALMT9 is a chloride channel, which is activated by physiological concentrations of cytosolic malate (De Angeli et al., 2013b). Based on its functional characteristics it has been proposed that this channel plays an important role in fast and complete opening of stomata, while having no effect on stomatal closure. Consistently, *Atalmt9* KO plants showed a drought resistant phenotype, which is in line with the findings of impaired stomatal opening due to decreased uptake of chloride in guard cells.

Electrophysiological characterization of vacuoles isolated from AtALMT6-GFP over expressing *Arabidopsis* plants revealed in patch clamp experiments large, calcium-activated, inward-rectifying malate currents, i.e., a malate flux from the cytosol to the vacuole. *Atalmt6* loss of function plants showed reduced malate currents in comparison to wild type *Arabidopsis* vacuoles. But presumably due to functional redundancy of malate transporters in guard cells, *Atalmt6* plants do not show phenotypic differences from the wild type (Meyer et al., 2011). AtALMT6 is regulated by changes in cytosolic Ca, being activated with increased levels in the micromolar range. Additionally, vacuolar pH and cytosolic malate are other key players of a regulatory mechanism imposing a threshold level for the activation of AtALMT6.

Expression analysis revealed also high expression of *AtALMT12* in guard cells. However, in contrast to AtALMT6 and AtALMT9, this protein is targeted to the guard cell plasma membrane. Comprehensive studies demonstrated AtALMT12 to function as a rapidly/quickly activating (R-type) anion channel (QUAC), which carries mainly chloride and nitrate currents (**Figure 1**) (Meyer et al., 2010b; Sasaki et al., 2010). The *Atalmt12* loss of function mutant showed impaired ABA, CO_2_, and dark-induced stomatal closure. These results were further supported by patch clamp analyses of guard cell protoplasts from the knock out plant. There, a diminished R-type current in the presence of malate in the bath medium was observed, when compared with the wild type. Functional expression of the AtALMT12 protein in *Xenopus* oocytes further confirmed its role as an R-type channel.

### Anion Homeostasis

The maize gene *ZmALMT1* encodes a plasma membrane-localized protein that, unlike its counterparts in wheat, *Arabidopsis*, and rape, is not involved in Al-activated organic acid exudation; it rather mediates anion influx and efflux. Transport of anions by ZmALMT1 is only feebly enhanced by Al and remains unchanged on changing internal citrate or malate concentrations (Piñeros et al., 2008b). Another member of the same family, ZmALMT2, is also localized on the plasma membrane and mediates large constitutive malate and citrate currents and is also permeable for the physiologically relevant anions Cl^-^ and NO_3_^-^. As ZmALMT1, it does not play a big role in Al tolerance response (Ligaba et al., 2012). Instead, both ZmALMT1 and ZmALMT2 are involved in plant nutrition and ion homeostasis (**Figure 1**). A similar role is played at the vacuolar level by AtALMT9 which mediates vacuolar malate uptake (Kovermann et al., 2007; Meyer et al., 2010a; Sasaki et al., 2010; De Angeli et al., 2013b).

### Fruit Quality

Sequestration of organic acids in fruits has a major impact on their taste, smell, and flavor, thereby strongly influencing their agronomic value. In apples, the varieties in flavor/acidity are determined by differences in accumulation of malic acid in the mature fruit. Genetic studies indicated that variation in fruit acidity is controlled by the major quantitative trait locus Ma (malic acid), which underlies an ALMT-like gene closely related to the *Arabidopsis* vacuolar AtALMT6 (Bai et al., 2012; Xu et al., 2012; Khan et al., 2013). Transient expression of Ma1::GFP in onion epidermal cells confirmed its vacuolar localization (**Figure 1**) (Ma et al., 2015). Likewise, the increased malic acid content in yeast cells overexpressing Ma1 is consistent with its putative role as an anion channel that mediates vacuolar malate accumulation *in planta*. Interestingly, the Ma locus consists of two different alleles, namely Ma1 and ma1, the latter coding for a truncated version of the protein due to a premature stop codon as a result of a single nucleotide substitution in the last exon. Further functional studies are required to figure out whether the allelic variants are functionally distinct, and thereby underlie the phenotypic differences in malic acid content.

**FIGURE 3:**
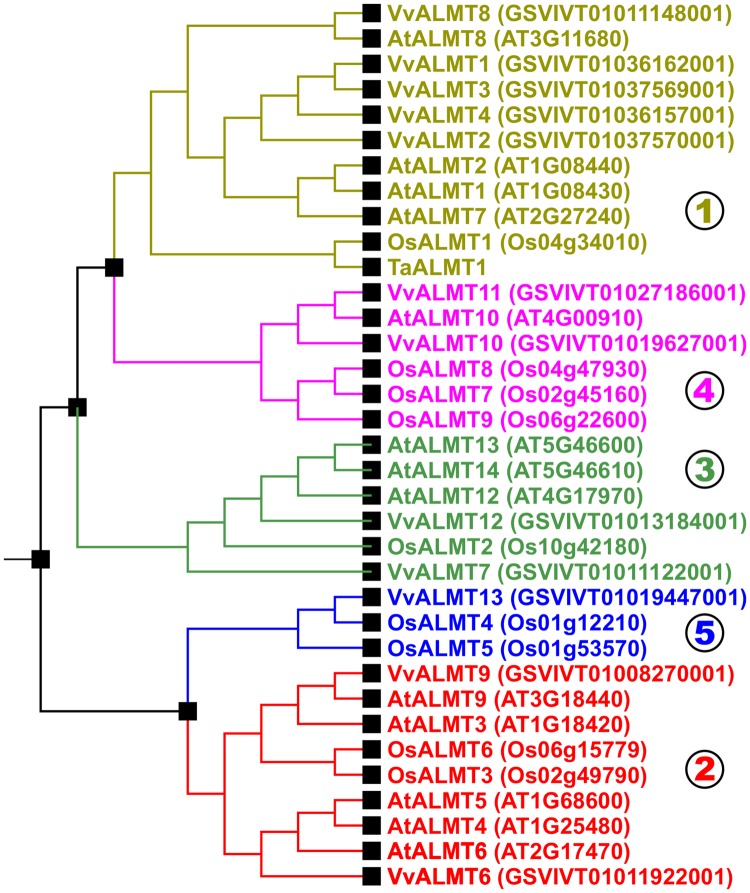
**Evolutionary relationship of ALMTs from *Arabidopsis thaliana*, *Oryza sativa* and *Vitis vinifera*.** ALMTs of higher plants segregate into five different clades. Figure adapted from Dreyer et al. (2012). Nomenclature of ALMTs from *V. vinifera* according to De Angeli et al. (2013a). For better illustration, only TaALMT1 from *Triticum aestivum* was included in the tree as an example for functionally characterized ALMTs from species different from *A. thaliana*, *O. sativa*, and *V. vinifera*. ZmALMT1, ZmALMT2, HvALMT1, ScALMT1, and HlALMT1 cluster with TaALMT1 and OsALMT1 in clade 1; BnALMT1 and BnALMT2 cluster with AtALMT1 in clade 1; GmALMT1 clusters with AtALMT8 and VvALMT8 in clade 1; and MdMA1 clusters with AtALMT4, AtALMT5, AtALMT6, and VvALMT6 in clade 2.

In grapes, the sequestration of organic acids, in particular malic acid and tartaric acid, is a key determinant for berry development and a deciding factor for wine quality and production (Conde et al., 2007). Phylogenetic analyses indicate the *Vitis vinifera* genome to contain 12 members of the ALMT family (**Figure 3**). Based on cellular localization studies of ALMT:GFP chimeras transiently expressed in tobacco leaves, De Angeli et al. (2013a) identified the vacuolar VvALMT9::GFP as a suitable AtALMT9 homolog that could potentially underlie the unidirectional movement of organic acids into grape berry vacuoles (**Figure 1**). *VvALMT9* is constitutively expressed in berry mesocarp tissue, and its expression is upregulated during fruit maturation. Electrophysiological analyses of vacuoles isolated from tobacco leaves transiently expressing the VvALMT9::GFP protein indicated that VvALMT9 mediates the selective flux of malate and tartrate into the vacuole. This suggests that VvALMT9 facilitates the accumulation of malate and tartrate in the vacuole of grape berries.

### Seed Development

HvALMT1 from barley shows large sequence similarity to TaALMT1 but it is not involved in Al tolerance. It is expressed in guard cells and mature root cells (Gruber et al., 2010). It is localized on the plasma membrane and unidentified motile vesicles in the cytosol mediating malate efflux and influx (**Figure 1**). Overexpression of HvALMT1 slows down the process of stomatal closure. These plants have a reduced growth rate in comparison to WT plants (Gruber et al., 2011). HvALMT1 helps in turgor regulations and balancing the osmoticum in expanding cells. Besides, it plays a crucial role in the acidification of the endosperm which is required for the activity of α-amylase, cysteine proteases, and ribonucleases that hydrolyze starch and storage proteins. HvALMT1 contributes to the release of malate in aleurone layers during seed germination (Xu et al., 2015).

### GABA – Receptor

**FIGURE 4:**
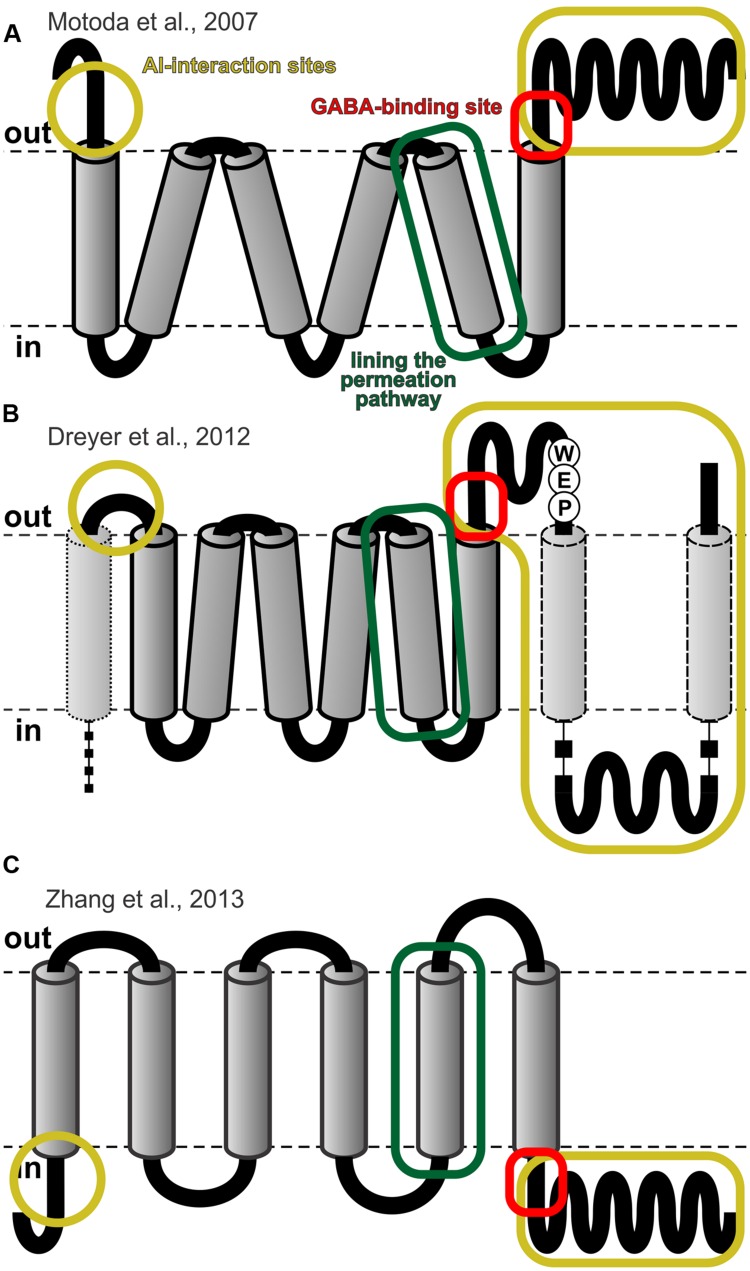
**Topological models for ALMTs. (A)** Topological model proposed by Motoda et al. (2007) based on immunocytochemical data. **(B)** Reconciliation of Motoda’s model with hydrophobicity analyses of the entire ALMT family and with experimental findings (Dreyer et al., 2012). **(C)** *Ad hoc* model proposed by Zhang et al. (2013) for vacuolar ALMTs by analogy with voltage-dependent K^+^ channels. Figures adapted from (Dreyer et al., 2012; Zhang et al., 2013). The frames in yellow indicate regions that are involved in Al-sensing (Ligaba et al., 2013; Sasaki et al., 2014), the frames in red the conserved GABA-binding site (Ramesh et al., 2015), and the frames in green the fifth transmembrane domain that was found to contribute to the permeation pathway (Zhang et al., 2013).

In an interesting study, Ramesh et al. (2015) showed that γ-aminobutyric acid (GABA) acts as negative regulator of TaALMT1, resulting in altered root growth and tolerance to Al, acidic, or alkaline pH. Stress conditions such as extreme pH, temperature, or salinity result in increased accumulation of GABA in plant tissues, which in turn prevents the release of malate from the root. This mechanism would help the plant to avoid excessive loss of reduced carbon which is crucial for plant growth and development under stress. Down-regulation of ALMT by GABA tends to hyperpolarize the membrane potential and therefore to decrease membrane excitability (Hedrich et al., 2016). Successful plant fertilization also needs a GABA gradient as it regulates growth of pollen tubes and directs it to the ovary. Upon analyzing other proteins of the ALMT family from different species, it was found that GABA also regulates anion-activated currents mediated by other members of this family. All ALMT proteins conserve a 12 aa long motif required for GABA regulation (**Figure 4**). This motif contains residues that have been associated with GABA binding in GABA_A_ receptors (Boileau et al., 1999). However, GABA-induced inhibition of ALMT protein activities is not completely abrogated by site-directed mutagenesis in this motif, hinting toward the existence of multiple GABA-binding sites. GABA fluxes are observed in and out of wheat roots, which appears to be futile but might be legitimized if GABA is essential for cell-to-cell communication and biotropic interactions (Gilliham and Tyerman, 2016).

### Microbe Interactions

Biotic factors, such as a pathogen attack, also regulate ALMT1 protein expression. In the study by Lakshmanan et al. (2012), a synthetic microbe-associated molecular pattern-peptide, flagellin 22 (flg 22) was found to induce AtALMT1 expression. A similar observation was made by Rudrappa et al. (2008), who showed increased release of malate via the induction of AtALMT1, when the aerial plant tissue was infected by pathogenic bacteria. As a consequence of the malate exudate, the plant defense mechanism strengthens by constituting a biofilm of beneficial bacteria at the root surface.

In *Lotus japonicus* the ALMT protein LjALMT4 is highly expressed in nitrogen fixing nodules. Heterologous expression of this protein in *Xenopus laevis* oocytes revealed that LjALMT4 mediates efflux of dicarboxylates. Since the protein is expressed in nodule vascular bundles, it is not involved in transport at the peribacteriod membrane, but is involved in efflux and influx of dicarboxylates and inorganic ions in Lotus nodule vasculatures (Takanashi et al., 2016).

## Structure – Function

### Molecular Evolution

The plant model organism *A. thaliana* has 14 genes that could be assigned to code for proteins belonging to the ALMT family. On the basis of amino acid sequence similarity these 14 members can be grouped into four different clades (**Figure 3**). Clade1 includes AtALMT1, 2, 7, and 8 (At1g08430, At1g08440, At2g27240, At3g11680), AtALMT10 (At4g00910) is member of clade 4, whereas clade 2 includes AtALMT3, 4, 5, 6, and 9 (At1g18420, At1g25480, At1g68600, At2g17470, and At3g18440). The protein family members AtALMT11, 12, 13, and 14 (At4g17585, At4g17970, At5g46600, At5g46610) belong to clade 3. It should be noted, however, that AtALMT11 is apparently not a full length protein. *AtALMT11* could be a truncated gene that does not encode a functional protein. In a larger evolutionary context, ALMTs are grouped into the Aromatic Acid Exporter (ArAE) family, which consists of bacterial and eukaryotic members from plants, yeast and protozoans (Transporter Classification Database 2.A.85^1^). The ALMTs, however, differ strongly from the non-plant members justifying the statement that ALMTs are unique to the plant kingdom. Systematic phylogenetic analyses showed that the ALMT family found today in higher plants can be subdivided into five clades (**Figure 3**) (Barbier-Brygoo et al., 2011; Dreyer et al., 2012). Clade 2/3 separated from clade 1/3/4 after the onset of bryophytes but before the appearance of lycophytes. After the emergence of lycophytes, first clade 3 separated from clade 1/4, then clade 1 and 4 diverged and finally clade 2 and 5 separated from each other (Dreyer et al., 2012). *Arabidopsis* does not have ALMTs that belong to clade 5.

### Secondary Structure and Topology

In the absence of a crystal structure that is suitable for generating homology models of ALMTs, their structural and topological analyses remain challenging and controversial. Sequence alignments and secondary structure predictions indicate that all members of the ALMT family share a high degree of structural similarity within the N-terminal half of the proteins, while the C-terminal half is more variable. Hydrophobic analyses suggest that the N-terminal part builds the hydrophobic core with 6–7 transmembrane domains (TMDs). The C-terminal part is mostly hydrophilic but may contain additional two TMDs or membrane-anchored domains (**Figure 4B**) (Dreyer et al., 2012). An earlier immunocytochemical study probing the topology of TaALMT1 suggested the transport protein to consist of six transmembrane domains, such that the N- and C-terminus face the extracellular side of the plasma membrane (**Figure 4A**) (Motoda et al., 2007). However, inconsistencies of this topology with the functional characteristics reported for some of the ALMT members [e.g., compare (Motoda et al., 2007; Ryan et al., 2011) with (Meyer et al., 2010b; Dreyer et al., 2012; Ligaba et al., 2013; Mumm et al., 2013)], as well as measurements of the pH-dependent fluorescence intensity of N- or C-terminally tagged ALMT:YFP chimeras expressed in *Xenopus* oocytes (Mumm et al., 2013) argue in favor of an opposite topology, where the N- and C-terminus face the cytoplasmic environment (**Figure 4C**) (Zhang et al., 2013).

Function-structure analysis aimed at characterizing changes in ALMT functionality upon structural modifications, including protein truncation, domain swapping, and single point mutations start to provide insight into the functional role of the N- and C-terminal domains (Furuichi et al., 2010; Ligaba et al., 2013; Mumm et al., 2013; Zhang et al., 2013; Sasaki et al., 2014). For example, a structurally modified TaALMT1 lacking the entire C-terminal region is functionally resembling the transport/permeation properties of the unmodified wild type, but lacks the Al-responsiveness that is typical of this transporter (Ligaba et al., 2013). These observations indicate that the TMD-containing N-terminal domain comprises and assembles the permeation pathway, whilst the hydrophilic C-terminal domain underlies regulatory properties. The regulatory nature of the C-terminus of AtALMT12 (also named as QUAC) has been shown to be a key determinant of the voltage-dependent gating of this channel (Mumm et al., 2013). However, in contrast to the voltage-independent TaALMT1, removal of the C-terminus in AtALMT12 hindered its functionality. Although these types of mutagenesis studies have provided a useful tool to probe structure-function relations of ALMTs, the interpretation of the outcomes should be treated cautiously, and within a phylogenetic context. For example, single point mutations of a glutamate residue (position 276 in TaALMT1 and 284 in AtALMT12, respectively) that is part of the characteristic WEP fingerprint motif (Trp-Glu-Pro) present in all ALMTs (Dreyer et al., 2012), results in total loss of electrogenic transport (Ligaba et al., 2013; Mumm et al., 2013). As exemplified by these studies, significant functional changes might result from alterations of family-wide structural integrity, rather than modification of residues specifically associated with a particular functional characteristic. Finally, biochemical approaches on AtALMT9 have proven the multimeric nature of the ALMTs suggesting that AtALMT9 putatively assembles as a tetramer (Zhang et al., 2013).

### Selectivity – Permeability

Functional analyses, for instance with electrophysiological techniques, have shown that members of the ALMT family are permeable to organic and inorganic anions and therefore mediate both their influx and their efflux. The malate permeability of TaALMT1 and the nomenclature (i.e., malate transporter) for this entire family of transporters was first established by two-electrode voltage-clamp experiments in *Xenopus* oocytes, whereby increasing the intracellular malate concentration experimentally by microinjecting malate resulted in an increase in inward current (i.e., anion efflux) in TaALMT1 expressing cells (Sasaki et al., 2004; Piñeros et al., 2008a). Various members of the ALMT family have also been shown to mediate efflux of inorganic anions when expressed in *Xenopus* oocytes, showing a general permeability sequence of NO_3_^-^ > Cl^-^, (Piñeros et al., 2008a,b; Ligaba et al., 2012; Sasaki et al., 2016). Patch clamp experiments, allowing access to ionic composition of both, the intracellular and extracellular environments of TaALMT1 expressed in tobacco protoplasts indicated that the inward current was highly selective to malate over nitrate and chloride, with a permeability sequence of mal^2-^ >> NO_3_^-^ >> Cl^-^ and a permeability ratio P_mal_/P_Cl_ larger than 18 (Zhang et al., 2008). Likewise, ion substitution experiments in *Xenopus* oocytes indicated a P_mal_/P_Cl_ between 10 and 30, depending on the external Cl^-^ concentration (Piñeros et al., 2008a). Noticeably, P_mal_/P_Cl_ as low as one have also been reported for other ALMT members [e.g., ZmALMT2 (Ligaba et al., 2012)]. Although various permeability/selectivity sequences have been reported for some ALMTs [e.g., malate > fumarate > Cl^-^ for AtALMT9 (Kovermann et al., 2007) and fumarate > malate >> citrate > Cl^-^ > NO_3_^-^ for ALMT6 (Meyer et al., 2011)], the relative permeability of a given anion (as well as the channel activity, see “Voltage-Dependence”) is highly dependent on the ionic composition of permeating anions on both sides of the membrane, and should therefore be expected to represent only an approximation of those expected under physiologically relevant ionic concentrations.

### Regulation

#### Enhancement by Extracellular Al^3+^

Several members of the ALMT family, including the family founder TaALMT1, have been associated with *in planta* responses to Al-stress (**Figure 1**). Typically, exposure to Al results both in an upregulation of genes encoding these transporters and a so called “Al-induced” release of organic acids to the rhizosphere. This “Al-induced” release of organic acids *in planta* is the product of either increased protein levels and/or a direct regulatory effect of Al^3+^ on the transporter. Investigations of various members of the ALMT family in heterologous expression systems indicate that only a few of them, namely TaALMT1, AtALMT1, and BnALMT1 are not just functionally active in the absence of extracellular Al^3+^, but also undergo conformational changes that lead to an enhancement of transport activity (i.e., malate release) upon exposure to extracellular Al^3+^. This direct Al-activation occurs with high affinity within 3–5 min; it is specific to Al, suggesting that it involves direct interaction of Al^3+^ with the ALMT protein (Sasaki et al., 2004; Hoekenga et al., 2006; Piñeros et al., 2008a; Zhang et al., 2008). The latter functional fingerprint, ambiguously referred to Al-activation, is the unfortunate basis for the existing nomenclature (i.e., ALMT: Al-activated malate transporter) of the entire family of transporters, as only these three ALMTs (and HvALMT to a smaller degree) have been reported to undergo “Al activation,” or rather Al^3+^-induced enhanced transport activity upon exposure to Al^3+^. These members are primarily localized on the plasma membrane of plant root cells; they are involved in the Al-regulated release of organic acids and underlie the far-reaching mechanism of Al-resistance in plant species. Along with Al, these proteins are also under tight regulation by different other factors. The current lack of suitable structural models has limited structural/functional and topological understanding of this transporter family (see “Secondary Structure and Topology”), hindering the understanding of the conformational changes undergone by the ALMTs upon interaction with Al^3+^. Nonetheless, several studies have started to elucidate the nature of some of the potential molecular interactions underlying this process. Neutralization of negatively charged residues in the C-terminal domain has abolished the Al-enhancement of the TaALMT1 protein (Furuichi et al., 2010; Ligaba et al., 2013), originally suggesting that a subgroup of residues (particularly Glu274, Asp275, and Glu284) were the key determinants of the transport enhancement process. However, in light of the uncertainty and the controversy regarding the exact topology of ALMTs, the uniqueness of these residues remains questionable. A detailed structure-function study cross-referenced with a phylogenetic analysis of the ALMT proteins, re-assessed the role of protein domains and negatively charged residues in terms of overall conservation and structural integrity, as well as their potential role in the molecular mechanism leading to the Al-dependent response (Ligaba et al., 2013). In this study, neutralization of negative residues throughout the entire TaALMT1 protein weakened or even abolished transport enhancement by Al^3+^. This study also highlighted the importance of integrating function-structure studies with a comprehensive phylogenetic analysis, as changes in protein functionality might result from alterations of residues involved in family-wide structural integrity, rather than the modification of residues specifically associated with a particular functional characteristic (e.g., transport enhancement by Al^3+^). Overall, these studies suggest that not only potential domains in the C-terminus, but rather domains present throughout the protein are likely to be involved in Al-mediated enhancement of transport activity. Another report support this inference by functionally characterizing ALMT chimeras, where N-terminal and C-terminal domains of Al-responsive ALMTs have been swapped (Sasaki et al., 2014).

#### Transcriptional Regulation

The promoter of the *AtALMT1* gene contains several cis acting elements. One of these is recognized by the transcription factor AtSTOP1 that is crucial for Al-induced higher expression of AtALMT1 (Iuchi et al., 2007; Tokizawa et al., 2015). Likewise, the expression of the *H. lanatus HlALMT1* gene is also regulated through the number of cis-acting elements in its promoter region that are targeted by the Al responsive transcription factor HIART1 (Chen Z.C. et al., 2013). In contrast to these positive regulators, the WRKY46 transcription factor acts as a negative regulator or repressor of AtALMT1. *wrky46* loss of function mutants show higher ALMT1 expression along with increased malate exudation resulting in better Al tolerance in mutant plants (Ding et al., 2013).

#### Voltage-Dependence

In spite of structural similarity, the members of the ALMT family show diverse current-voltage (I-V) relationships. The voltage-dependent properties of ALMTs are best described so far for AtALMT12 (clade 3), and AtALMT6, AtALMT9, and VvALMT9 (all clade 2). AtALMT12 is open at positive voltages and closes with hyperpolarization. Upon voltage steps, current amplitudes relax within ~100 ms into a new voltage-dependent equilibrium (Meyer et al., 2010b; Imes et al., 2013; Mumm et al., 2013). The vacuolar channels AtALMT6, AtALMT9, and VvALMT9 are more active at negative voltages than at positive; they need several seconds to reach a new steady state after a voltage-step (Kovermann et al., 2007; Meyer et al., 2011; De Angeli et al., 2013a). Channels belonging to clade 1, in contrast, do not show pronounced signs of voltage-dependence. Upon voltage-steps, the current follows instantaneously the altered driving force (Sasaki et al., 2004; Hoekenga et al., 2006; Piñeros et al., 2008a,b; Ligaba et al., 2012). In some cases (e.g., ZmALMT1, TaALMT1, GmALMT1), a slight voltage-dependent inactivation of the currents is notable at sustained hyperpolarization. The reason for the voltage-dependence of some ALMTs is rather unclear; these channels do not comprise a region with clustered charges that may serve as a voltage-sensor. Instead, for AtALMT12 it was shown that the C-terminal end of the channel is involved in voltage-dependent gating. Fusion of GFP to the C-terminus rendered this channel voltage-insensitive. Therefore, it is speculated that a flexible C-terminus might block the channel in a “ball at a chain” manner (Mumm et al., 2013). Additionally, ALMTs react on intracellular and extracellular (or vacuolar) conditions. Detailed analysis of tonoplast-localized AtALMT6 showed regulation of channel activity through pH and cytosolic malate. These two factors determine whether ALMT6-conducted currents would be inwardly or outwardly rectifying depending on the vacuolar membrane potential (Meyer et al., 2011). In particular, the permeating ion malate increases channel activity from the cytosolic and from the extracellular (or vacuolar) site (De Angeli et al., 2013a,b). Also guard cell R-type anion channel activity is modulated by permeating anions on both sides of the membrane (Hedrich and Marten, 1993, Hedrich et al., 1994; Dietrich and Hedrich, 1998; Frachisse et al., 1999; Diatloff et al., 2004). It is thus speculated that ALMTs comprise two independent (so far not identified) malate-binding sites that contribute to the voltage-sensing process (Mumm et al., 2013).

#### Nucleotides/Phosphorylation

An apparent voltage-dependence of ALMTs can also be caused by cytosolic nucleotides that modulate/inhibit the channel activity by a reversible block of the pore region in a voltage-dependent manner (Zhang et al., 2014). Regulation of guard cell anion channels by cytosolic nucleotides has been reported previously (Hedrich et al., 1990; Schulz-Lessdorf et al., 1996; Thomine et al., 1997). Cytosolic nucleotides are centrally involved as substrates in phosphorylation processes that regulate channel activity. However, further analysis of R-type/QUAC channels proposed also a direct effect of cytosolic nucleotides as a ‘voltage-dependent gate’ that blocks the channel pore at hyperpolarized potentials (Colcombet et al., 2001). Thinking along the same line, the I-V relationship of AtALMT9 was studied and it was found that the channel is also regulated by cytosolic nucleotides. The block of AtALMT9 in the presence of ATP is voltage-dependent and it changes the monotonic I-V curve into a bell-shaped curve. The non-hydrolysable ATP analog AMPPNP was found to induce an even stronger inhibitory effect as compared to ATP confirming that the block of the channel activity is due to direct occlusion of the permeation pathway and not a secondary effect of phosphorylation. Mutation of Lys-193 in the putative pore region of the channel completely nullifies the effect of cytosolic nucleotides. The mechanism of voltage gating in plasma membrane and vacuolar channels based on the physical occlusion of the channel permeation pathway occurs at physiological ATP concentration at negative resting potentials and is orchestrated by the anion concentrations on both sides of the membrane (Zhang et al., 2014; De Angeli et al., 2016).

Cytosolic nucleotides appear to exert a bimodal regulation on ALMTs. Besides serving as voltage-dependent blockers they are essential as co-factors of kinases that phosphorylate and activate these channels (Ligaba et al., 2009; Imes et al., 2013). The activity of AtALMT12/QUAC1 is regulated by phosphorylation through the kinase Open Stomata 1 (OST1), which in turn is activated by ABA under stress conditions. Patch clamp studies on guard cells from the wild type and an *ost1* loss of function mutant show activation of R-type/ALMT12 currents in the wild type, which is considerably diminished in *ost1*. Co-expression of ALMT12 and OST1 in *Xenopus* oocytes resulted in a noticeable increase in R-type/ALMT12 activity (Imes et al., 2013). In the wheat channel TaALMT1 the amino acid S384 was identified as key residue for regulating channel activity via direct protein phosphorylation (Ligaba et al., 2009), a finding that fueled the discussion on the topology of ALMTs (**Figure 4**) (Motoda et al., 2007; Dreyer et al., 2012).

## Conclusion

The historical protein family name “ALMT” could be largely misleading as meanwhile it is clear that many ALMTs are not involved in aluminum tolerance. Their physiological roles go far beyond, and up to now we have just got a small glimpse on this diversity. Future research will certainly be boosted when reliable structural data are available that allow to correlate structural motifs with functional properties. We should be prepared for more surprises from this protein family.

## Author Contributions

All authors conceived the project and had intellectual input on the project. TS, ID, and MP wrote the manuscript. All authors commented on the manuscript.

## Conflict of Interest Statement

The authors declare that the research was conducted in the absence of any commercial or financial relationships that could be construed as a potential conflict of interest.

## Footnotes

1 http://www.tcdb.org/
